# Heparin interacts with elongation factor 1α of *Cryptosporidium parvum* and inhibits invasion

**DOI:** 10.1038/srep11599

**Published:** 2015-07-01

**Authors:** Atsuko Inomata, Fumi Murakoshi, Akiko Ishiwa, Ryo Takano, Hitoshi Takemae, Tatsuki Sugi, Frances Cagayat Recuenco, Taisuke Horimoto, Kentaro Kato

**Affiliations:** 1 Department of Veterinary Microbiology, Graduate School of Agricultural and Life Sciences, The University of Tokyo, 1-1-1 Yayoi, Bunkyo-ku, Tokyo 113-8657, Japan; 2 National Research Center for Protozoan Diseases, Obihiro University of Agriculture and Veterinary Medicine, Inada-cho, Obihiro, Hokkaido 080-8555, Japan

## Abstract

*Cryptosporidium parvum* is an apicomplexan parasite that can cause serious watery diarrhea, cryptosporidiosis, in human and other mammals. *C. parvum* invades gastrointestinal epithelial cells, which have abundant glycosaminoglycans on their cell surface. However, little is known about the interaction between *C. parvum* and glycosaminoglycans. In this study, we assessed the inhibitory effect of sulfated polysaccharides on *C. parvum* invasion of host cells and identified the parasite ligands that interact with sulfated polysaccharides. Among five sulfated polysaccharides tested, heparin had the highest, dose-dependent inhibitory effect on parasite invasion. Heparan sulfate-deficient cells were less susceptible to *C. parvum* infection. We further identified 31 parasite proteins that potentially interact with heparin. Of these, we confirmed that *C. parvum* elongation factor 1α (CpEF1α), which plays a role in *C. parvum* invasion, binds to heparin and to the surface of HCT-8 cells. Our results further our understanding of the molecular basis of *C. parvum* infection and will facilitate the development of anti-cryptosporidial agents.

The genus *Cryptosporidium* is an intracellular parasite in apicomplexan^1^, which comprises 30 species classified based on their host specificity^2^. *Cryptosporidium parvum* can cause serious watery diarrhea in human and other mammals^3^. Most immuno-competent individuals experience self-limited diarrhea that resolves within 1–2 weeks^4^; however many immuno-compromised patients, such as patients with AIDS, can experience prolonged diarrhea that becomes life-threatening^4^.

*C. parvum* is also a problem in the farming industry; it is one of the most common enteropathogens in young calves, sheep, and goats^3^. Although most infected calves are asymptomatic in the absence of co-infection with viruses, bacteria, or other parasitic pathogens, cryptosporidiosis still causes economic losses for farmers^5^.

To date, several substances, including paromomycin, nitazoxanide, and lasalocid, have been shown to have anti-cryptosporodial activity. Paromomycin, an aminoglycoside, reduces oocyst excretion and stool frequency in immuno-compromised patients^6^. Nitazoxanide is only the drug approved by the FDA^7^ for the treatment of cryptosporidiosis; it effectively eradicates *C. parvum* oocysts from stool and resolves the diarrhea of both immuno-competent and -compromised individuals^8^. Lasalocid is an ionophorous antibiotic that reduces oocyst excretion in cattle^9^,^10^. However, all of these agents have limited effects on parasite growth, highlighting the urgent need for novel effective anti-cryptosporodial drugs.

*C. parvum* infection occurs when a host animal ingests *C. parvum* oocysts^11^. The oocysts undergo excystation and release sporozoites while passing through the stomach and duodenum due to the rise of temperature, bile salt, and digestive enzymes^12^. These sporozoites attach to intestinal epithelial cells, subsequently are embraced by the host cell membrane and develop into trophozoites in epicellular space^13^. This invasion is a critical step for the development of disease caused by *C. parvum*, yet there have been relatively few studies on the molecular basis of the host-parasite interactions that are necessary for *C. parvum* invasion of host cells^12^. We do know that Gal/GalNac is recognized by *C. parvum* p30^14^, and that the 85-kDa protein on Caco-2 cells is a receptor for *C. parvum* circum sporozoite-like antigen^15^. But the remaining host factors that interact with *C. parvum* sporozoites at the invasion step remain largely unknown.

*C. parvum* infects mainly the gastrointestinal tract^16^. However, sporozoites cannot directly interact with intestinal epithelial cells because glycocalyx, a filamentous layer of branched carbohydrates^17^, is present on these cells and act as a defensive barrier. Glycocalyx contains high levels of transmembrane mucin glycoproteins^18^, a type of proteoglycan. Mucin is the major component of the intestinal barrier^19^ and has been reported to reduce *C. parvum* attachment to intestinal epithelial cells *in vitro*^20^.

Proteoglycans (PGs) are composed of glycosaminoglycan (GAG) chains, which is a category of sulfated polysaccharides that are covalently bound to a protein core^21^. A variety of pathogens utilize them to invade their host cells^22^,^23^,^24^. Some sulfated polysaccharides have been shown to bind to several apicomplexan parasites, including *Toxoplasma gondii* and *Plasmodium falciparum,* and to inhibit their infection^25^,^26^,^27^,^28^,^29^. Therefore, we hypothesized that *C. parvum* interacts with GAGs on the host cells and that some sulfated polysaccharides may inhibit *C. parvu*m infection. Despite many reports about the inhibitory effects of sulfated polysaccharides on various apicomplexan parasites, little is known about the interaction between *C. parvum* and sulfated polysaccharides^14^,^30^.

In this study, we evaluated the anti-cryptosporidial effects of five sulfated polysaccharides on parasite invasion, and found that heparin had the highest inhibitory effect on the *C. parvum* invasion of host cells. To gain further insight into the heparin-induced inhibitory mechanism of parasite invasion, we subsequently attempted to identify the *C. parvum* sporozoite proteins that physically bind to heparin.

## Results

### The effects of sulfated polysaccharides on *C. parvum* invasion of host cells

**Figure 1:**
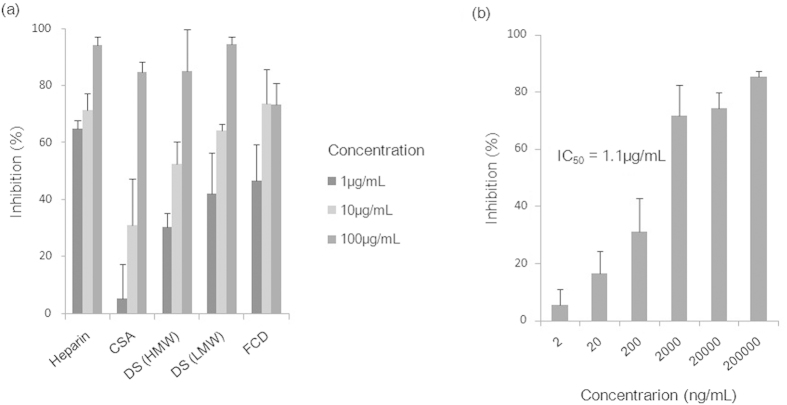
Inhibitory effects of sulfated polysaccharides on *C. parvum* sporozoite invasion of HCT-8 cells. *C. parvum* sporozoites were inoculated to HCT-8 cells in RPMI-1640 medium containing each sulfated polysaccharide. The number of parasites left in the HCT-8 cells was counted per 100 fields of view by use of fluorescence microscopy. Each assay was performed in independent triplicates, and means ± standard deviations are shown. (**a**) Inhibitory efficacy of sulfated polysaccharides. Each sulfated polysaccharide was added at the concentration of 1, 10, or 100 μg /mL in RPMI-1640 medium. (**b**) Inhibitory efficacy of heparin tested over a wide range of concentrations. Heparin was added at the concentrations of ten-fold serial dilutions from 2,000,000 to 2 ng/mL in RPMI-1640 medium.

To examine the effects of sulfated polysaccharides on the invasion of host cells by *C. parvum*, we performed invasion inhibition assays with the following five sulfated polysaccharides: heparin, chondroitin sulfate A (CSA), dextran sulfate [DS high molecular weight (HMW) and DS low molecular weight (LMW)], and fucoidan (FDC) (Fig. 1A). All of these polysaccharides showed dose-dependent inhibitory effects on the *C. parvum* invasion of HCT-8 cells, inhibiting over 50% of invasion at a concentration of 100 μg/mL; however, the inhibitory effect differed among the polysaccharides. Of the five polysaccharides tested, heparin inhibited the most *C. parvum* invasion at the lower concentration of 1 μg/mL and its inhibitory effect was dose independent (Fig. 1B). Our data reveal that sulfated polysaccharides differ in their invasion inhibitory effects, with heparin having the highest and dose-dependent inhibitory effect on *C. parvum* invasion of HCT-8 cells among the sulfated polysaccharides tested. This result revealed that heparin competed with some factor(s) involved in the HCT-8 cell-parasite interaction *in vitro*.

### *C. parvum* sporozoites are affected by heparin

**Figure 2:**
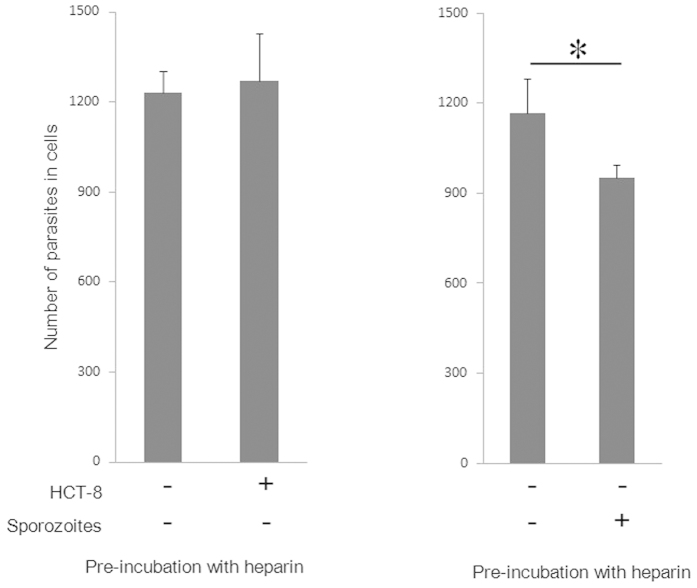
Inhibitory effects of heparin pre-incubated with sporozoites or HCT-8 cells. To determine whether heparin affects *C. parvum* sporozoites or HCT-8 cells, a pre-incubation assay was conducted. The both panels show the number of parasites that invaded HCT-8 cells. HCT-8 cells pre-incubated with heparin prior to *C. parvum* infection showed no reduction in parasite invasion (left panel), whereas HCT-8 cells inoculated with *C. parvum* that had been pre-incubated with heparin showed a statistically significant decrease in parasite invasion (~18%) (right panel). Each assay was performed in independent triplicate, and means ± standard deviations are shown. Statistically significant differences in the number of parasites in the cells were determined by using the Welch’s T-test; *P* values less than 0.05 are shown by the asterisk.

To understand the mechanistic basis of the inhibitory effect of heparin on the invasion of culture cells by *C. parvum*, we conducted pre-incubation assays to elucidate whether heparin affects HCT-8 cells or parasites. No invasion inhibition was observed in the HCT-8 cells incubated with heparin prior to parasite infection (Fig. 2, left panel), whereas significant inhibition was observed in cells infected with parasites before incubation with heparin (Fig. 2, left panel). These results suggest that heparin competed with some factor(s) on the parasites rather than the HCT-8 cells, and that these parasites’ factors are involved in the invasion of HCT-8 cells *in vitro*.

### Heparan sulfate is important for the efficient invasion by *C. parvum*

**Figure 3:**
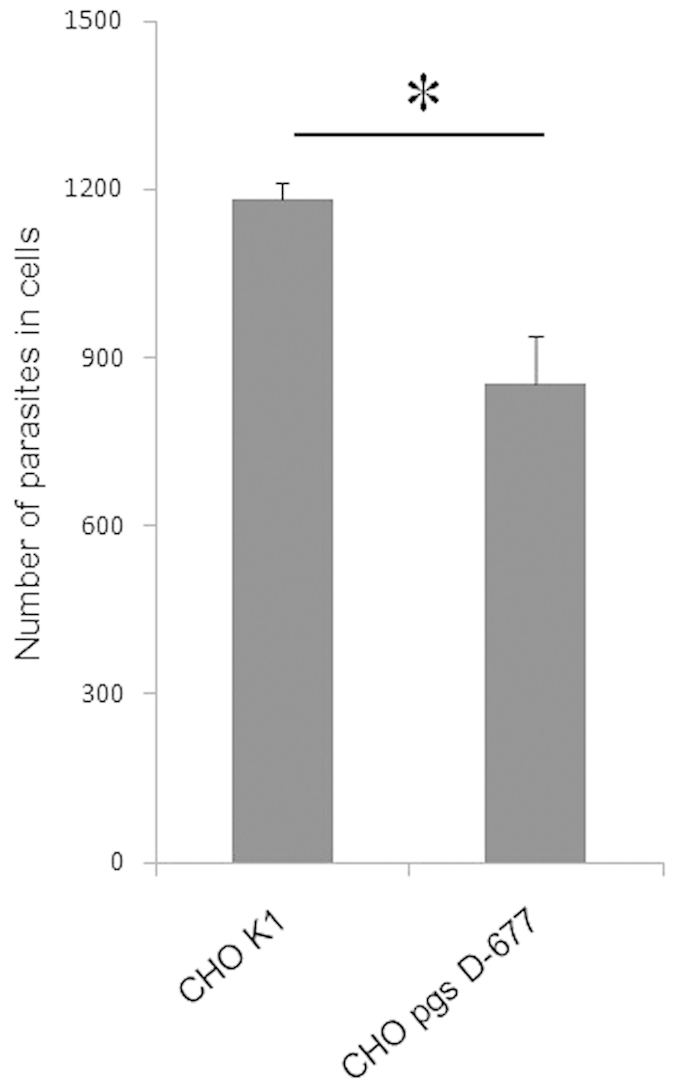
Heparin-deficient CHO cell lines are less susceptible to *C. parvum* infection. The invasion inhibition assay was also conducted using CHO K1 and CHO pgsD-677 strains. The number of parasites that invaded these cells is shown for each cell line. CHO pgs D-677 cells were less susceptible to *C. parvum* infection by 27% than were CHO K1 cells. Each assay was performed in independent triplicate, and means ± standard deviations are shown. Statistically significant differences in the number of parasites in the cells were determined by using the Welch’ s T-test; *P* values less than 0.05 are shown by the asterisk.

To determine whether heparin or heparin-like molecules are involved in the invasion of by *C. parvum* sporozoites, we compared the efficiency of parasite invasion of CHO pgs-D677 with that of wild-type CHO K1 cells. CHO pgsD-677 cells lacks both N-acetylglucosaminyl- and glucuronosyl-transferase, which are enzymes required for the polymerization of heparan sulfate or heparin^31^. Notably, heparin is mast cell polysaccharide^32^. Therefore, CHO pgsD-677 cells differ from CHO K1 cells in that they lack heparan sulfate, which is a kind of GAG and has a very similar structure to that of heparin. Cells were infected with *C. parvum* sporozoites, and the number of cells invaded by the parasites was compared. CHO pgsD-677 cells showed a statistically significant reduction in parasite invasion (27%) compared with that observed in wild-type CHO cells (Fig. 3). Thus, we demonstrated that heparan sulfate on the cell surface plays a role in invasion by *C. parvum in vitro*.

### Identification of *C. parvum*-derived factors that interact with heparin

**Figure 4:**
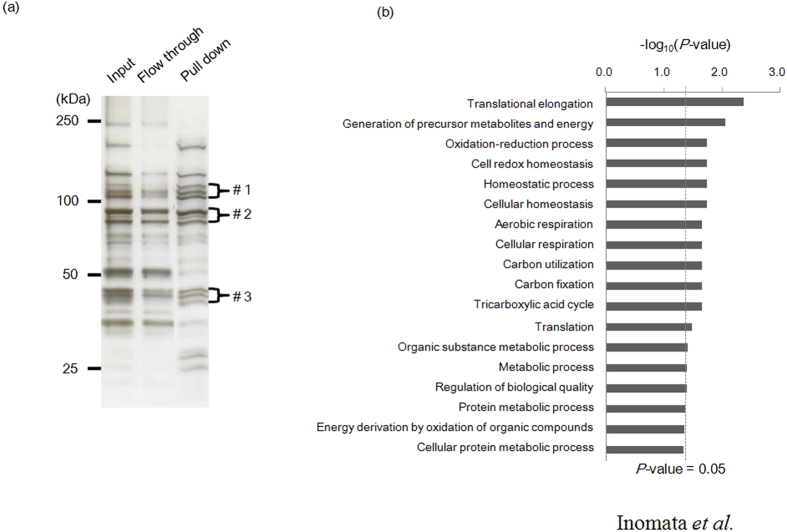
Identification of heparin-binding proteins of *C. parvum* sporozoites. (**a**) Silver staining showing the whole lysates of *C. parvum* sporozoite (lane 1, Input), heparin-unbound proteins (lane 2, Flow through), and heparin-binding proteins (lane 3, Pull down). The proteins in the bands with molecular masses of 120 (#1), 90 (#2), and 45 (#3) kDa were specifically concentrated in the precipitated fractions, and proteins in these three bands were separately gel-extracted and subjected to mass spectrometry analysis. (**b**) Gene enrichment analysis of heparin-binding proteins. To functionally categorize the proteins that interacted with heparin, all of the proteins identified by mass spectrometry were assigned to a GO grouping. GO analysis was carried out by using Gene Ontology Enrichment embedded in the CryptoDB database (http://cryptodb.org/), where Fisher’s exact *P* values were used to determine the GO terms that were statistically significant (*P* < 0.05).

To identify parasite factors that interact with heparin, we conducted pull down assays with cell lysates of *C. parvum* sporozoites by using heparin-agarose beads, and subsequently analyzed the precipitated parasite proteins by using liquid chromatography tandem mass spectrometry (LC-MS/MS). We found bands specifically concentrated with molecular masses of around 120, 90, and 45 kDa in the precipitated fraction (Fig. 4A). The proteins in these three bands were gel-extracted and subjected to mass spectrometry analysis. For the heparin-binding proteins, we identified a total of 31 distinct proteins; 7 of these proteins were detected in the band with the molecular mass of 120 kDa, 11 proteins were detected for 90 kDa, and 13 for 45 kDa (Table 1).

To further categorize these proteins based on molecular function, we performed functional enrichment analysis by using Gene Ontology (GO) analysis. These 31 proteins were enriched for multiple biological processes, that is, translation, homeostasis, metabolism, and respiration (Fig. 4B). Of note, the most enriched GO category for the heparin-binding proteins was translational elongation (*P* value = 4.1 × 10^−3^), including elongation factor 1 alpha (EF1α, cgd6_3990) and translation elongation factor 2 (EF2, cgd8_2930). Thus, we identified 31 parasite proteins with the potential to interact with heparin that are involved in diverse biological processes.

### *C. parvum* elongation factor 1α (CpEF1α) is a heparin ligand

**Figure 5:**
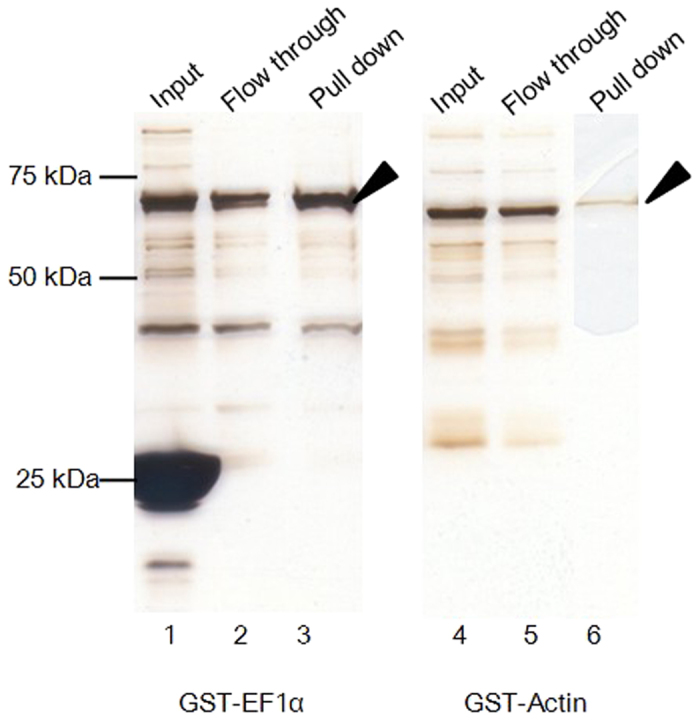
Heparin-binding property of rCpEF1α. To examine whether recombinant CpEF1α binds to heparin, a pull down assay was conducted. Purified GST-CpEF1α and GST-CpActin were incubated with heparin agarose beads (Input; lanes 1 and 4), and beads were then pulled down. Heparin-unbound phase and heparin-binding proteins are shown for GST-CpEF1α (Lanes 2 and 3), and for GST-CpActin (Lanes 5 and 6), respectively. Arrows indicate GST-fused recombinant proteins.

Among the parasite proteins that precipitated with heparin-agarose beads, *C. parvum* EF1α (CpEF1α) and EF2 belong to the most enriched GO category. Matsubayashi, M. *et al.* have previously showed that CpEF1α plays a role in the invasion and could be a potential protective antigen of *C. parvum*^33^, yet its host receptor has not been identified. We focused on CpEF1α and tested whether it directly interact with heparin. The GST-fusion CpEF1α was expressed at the expected molecular weight of 74 kDa in *E. coli*, was purified by GST beads, incubated with heparin-agarose beads, and its binding was assessed by silver staining. Actin was selected as a negative control because it did not belong to any GO categories and is often detected by mass spectrometry due to its abundance as a house-keeping gene. As we expected, a 68-kDa protein representative of GST-fused CpActin was scarcely pulled down with heparin-agarose beads (Fig. 5, right panel). By contrast, GST-fused CpEF1α was precipitated with heparin-agarose beads and a clear band was observed at the 74 kDa position (Fig. 5, left panel). Thus, we demonstrated that CpEF1α directly binds to heparin.

**Figure 6:**
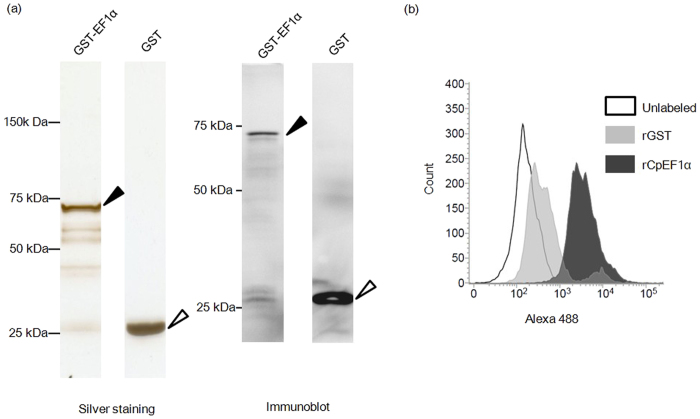
HCT-8 binding property of rCpEF1α. (**a**) The purified recombinant proteins were analyzed by SDS-PAGE and immunoblotting. The recombinant proteins were expressed in *E. coli* and purified by GST affinity chromatography. The purified proteins were concentrated by ultra-filtration. The black and white arrows indicate rCpEF1α and rGST, respectively. (**b**) Flow cytometry of HCT-8 cells incubated with recombinant protein. HCT-8 cells were incubated with each recombinant protein, reacted with rabbit α-GST antibody IgG, stained with Alexa 488-conjugated α-rabbit IgG goat antibody, and then subjected to flow cytometry analysis. The cells were gated on forward/side-light scatter to distinguish them from debris. The cells incubated with rCpEF1α (dark gray-filled histogram) showed higher fluorescence intensity than those incubated with rGST (bright gray-filled).

Finally, to investigate the biological interaction between CpEF1α and the receptor on the surface of the HCT-8 cell, the number of HCT-8 cells that binds to rCpEF1α was counted by using flow cytometry. Prior to binding assay, recombinant rCpEF1α and rGST was purified by ultrafiltration and the expression of the recombinant proteins were confirmed by silver staining and immunoblot. rGST served as a negative control. The HCT-8 cells incubated with rCpEF1α showed more fluorescence intensity than that observed for cells incubated with rGST (Fig. 6B). We observed statistically significant differences in the fluorescence intensity between the cells incubated with rGST and those incubated with rCpEF1α (*P *< 0.05), demonstrating that rCpEF1α binds to HCT-8 cells.

## Discussion

Here, we evaluated the inhibitory effect of sulfated polysaccharides on the invasion of HCT-8 cells by *C. parvum*, and found that heparin was the most effective inhibitor of *C. parvum* invasion among the five sulfated polysaccharides tested. To our knowledge, this is the first report of the effect of heparin on *C. parvum* invasion. We also showed that heparin competes with some factor(s) involved in *C. parvum* sporozoite invasion. In addition, we showed that heparin does not affect the HCT-8 cells but rather the *C. parvum* sporozoites. We further identified 31 parasite proteins that interact with heparin by using pull-down assays followed by mass spectrometry. We confirmed the binding of CpEF1α with a heparin-like molecule on the surface of HCT-8 cells. Taken together, our data suggest that a heparin-like molecule is important for the efficient invasion of HCT-8 cells by *C. parvum* sporozoites.

We observed a discrepancy in the inhibition efficacy of parasite invasion by heparin between the invasion inhibition assay and the pre-incubation assay. While 65% of parasite invasion was blocked by 1 μg/mL heparin in the invasion inhibition assay, only 18% was blocked in the pre-incubation assay. This discrepancy could be attributed to the amount of heparin present in the medium. In the former experiment, heparin was abundant in the medium and bound to sporozoites when the sporozoites were inoculated. By contrast, in the latter experiment, only bound heparin was present because the sporozoites were washed three times after pre-incubation with heparin and then inoculated. In other words, the free heparin present in the medium might have contributed to the inhibition of parasite invasion of host cells. Heparin may, therefore, bind to two different types of *C. parvum* sporozoite factor: surface proteins of sporozoites and secreted proteins involved in the invasion of the host cells by the sporozoites, resulting in efficient invasion inhibition.

The CHO pgsD-677 cell line was used as a heparan sulfate-deficient cell in our study. This cell line was less susceptibility to *C. parvum* sporozoite invasion than were wild-type cells. This cell line, however, expresses 3- to 4-fold higher levels of chondroitin sulfate than do wild-type cells^31^. Despite these higher levels of chondroitin sulfate, this cell line was much less susceptible to parasite invasion (Fig. 1A), indicating that heparan sulfate plays an important role in *C. parvum* invasion.

In our experiments, heparin did not completely inhibit *C. parvum* infection. Additionally heparan sulfate-deficient cells were less susceptibility to *C. parvum.* These results suggest heparan sulfate is not the sole receptor of *C. parvum*. Namely, other factors could cooperate to attach and invade into host cells. However, heparan sulfate is common molecule to mammals and this could help *C. parvum* is parasitic on many kinds of mammals. Therefore, heparan sulfate could be important molecule for infection and heparin is noticeable substance.

Eukaryotic EF1α (eEF1α) is involved in the first step of translation and elongation by binding and delivering aa-tRNAs to the A site of the ribosome^34^. In addition, eEF1-α has a multipleother functions^35^, including interaction with the actin cytoskeleton^36^ and modulation of microtubules in a Ca^2+^/CaM-dependent manner in mammalian cells^37^. By contrast, CpEF1α is reported to localize to the apical region of sporozoites, and play an essential role in invasion of sporozoites^33^. However, the binding partner of CpEF1α has not yet known. In the present study, we identified heparin as a CpEF1α-binding partner, and confirmed that recombinant CpEF1α bound to heparin and HCT-8 cells *in vitro*. Our results thus further our understanding of host-parasite interactions that are essential for parasite invasion of host cells.

Studies of various pathogens, including parasites^27^,^38^,^39^,^40^, viruses^41^,^42^,^43^, and bacteria^44^,^45^,^46^, strongly indicate that heparin binds to a range of microorganism proteins. Furthermore, heparin is known to inhibit the infection of various kinds of pathogen^41^,^43^,^47^,^48^. Most of the existing anti-cryptosporidial medicines are antibiotics or ionophores^6^,^7^,^8^,^9^,^10^. But these medicines have side effects and there is little information about safe dosages. Our study showed that heparin has an inhibitory effect on *C. parvum* infection. Although heparin is a sulfated polysaccharide, it can be synthesized in *Escherichia coli*^49^,^50^. Therefore, heparin could serve as a new type of anti-cryptosporidial agent. For clinical use, experimental testing in both humans and livestock is necessary. In addition, there are potential concerns regarding drug delivery and side effects. Ingested heparin could be digested in the stomach and not reach the small intestine. Therefore, it may be necessary to protect the heparin molecule with some kind of capsule. Moreover, heparin has anticoagulant effects^51^, suggesting that it could affect the blood coagulation system if ingested as a drug. A possible plan to utilize heparin is employing chemically-modified heparins^52^ which exhibit attenuated anticoagulant activity but, which keep an ability to inhibit *C. parvum* infection. If this substance is made practical, our research will contribute to progression of the treatment for cryptosporidiosis.

In summary, we revealed that heparin inhibits *C. parvum* invasion of host cells. We identified CpEF1α as a heparin-binding protein and characterized its heparin-binding property and affinity to HCT-8 cells. These results suggest that CpEF1α interacts with heparan sulfate on host cells and that this interaction is important for host cell invasion. Our findings help further our understanding of the molecular basis of *C. parvum* invasion and are of value for the development of novel anti-cryptosporidial agents.

## Methods

### Cells and parasites

Human ileocecal colorectal adenocarcinoma (HCT-8) cells (obtained from the American Type Culture Collection (ATCC), VA, USA) were maintained in RPMI-1640 medium (Sigma-Aldrich, MO, USA) supplemented with 10% fetal bovine serum, 2 mM L-glutamine, 15 mM HEPES, 50 units/mL penicillin, and 50 μg/mL streptomycin. Wild-type CHO-K1 cells, and CHO D-677 cells (obtained from ATCC) were maintained in Ham’s F-12 medium (Invitrogen, CA, USA) supplemented with 10% fetal bovine serum, 50 units/mL penicillin, and 50 μg/mL streptomycin. All cultures were maintained at 37 °C in a humidified atmosphere containing 5% CO_2_.

*C. parvum* oocysts, strain HNJ-1, were kindly provided by Dr. K. Yagita (National Institute of Infectious Diseases). Oocysts were maintained by passage in experimentally infected nude mice (SLC, Shizuoka, Japan) and purified from feces by using discontinuous sucrose and cesium chloride gradients as described previously^53^.

### Sporozoites excystation

*C. parvum* oocysts were treated with 10% (v/v) purelox (OyaloxCo.Ltd., Tokyo, Japan) for 10 min on ice, and then washed three times with phosphate buffered saline (PBS) by centrifugation at 7,000 rpm for 2 min at 4 °C. Purelox-treated and washed oocysts were treated with 0.1 M NaH_2_PO_4_-HCl (pH 2.0) for 30 min at 37 °C and then washed twice. The oocysts were further excysted in the PBS containing 0.75% sodium taurocholate and 0.25% trypsin for 1 h at 37 °C. Excysted sporozoites were separated from oocysts by filtration through a 5-μm pore-size PVDF filter (Merck Millipore, Darmstadt, Germany) and then used in the study. Oocysts and sporozoites were counted with microscopy using hemocytometer.

### Infection inhibition assay

The following five sulfated polysaccharides were used in this study: heparin, chondroitin sulphate A (CSA), dextran sulfate [Molecular weight: 6.5–10 kDa (DS (LMW)], dextran sulfate [MW > 50 kDa (DS (HMW)], and fuccoidan (FCD). These polysaccharides were purchased from Sigma-Aldrich. A total of 2.5 × 10^5^ HCT-8 or CHO cells were seeded in an 8-well chamber slide (Thermo Fisher Scientific Inc., MA, USA) and incubated overnight. The cells were then infected with 2.5 × 10^6^ *C. parvum* sporozoites in RPMI-1640 medium containing each sulfated polysaccharide for 3 h. Cells were washed with the medium three times, and further incubated for 3 h. The cells were washed with PBS three times and then stained with Sporo-Glo (Waterborne Environmental, Inc., VA, USA) after fixation with ice-cold 100% methanol, and the number of parasites left in the HCT-8 cells or CHO cells was counted per 100 fields of view with 400 magnification by means of fluorescence microscopy using Axio Vert.A1 (Zeiss, Oberkochen, Germany).

### Pre-incubation assay

*C. parvum* sporozoites or HCT-8 cells were incubated in RPMI-1640 medium containing heparin for 1 h prior to infection. Sporozoites or HCT-8 cells were washed with the medium without heparin three times, and subsequently mixed with HCT-8 cells or sporozoites, respectively, for 3 h. The number of parasites left in the cells was counted by following the protocol described above.

### Plasmids

3.0 × 10^8^ *C. parvum*, strain HNJ-1, sporozoites were used for DNA extraction. The sporozoite genomic DNA was extracted by using the QIAamp DNA Micro Kit (QIAGEN, Venlo, Netherlands) according to the manufacturer’s instructions. CpEF1α open reading frame was amplified by PCR with a pair of primers [forward, CpEF1-α-EcoRI-F (5’-ACT GAA TTC ATG GGT AAG GAA AAG ACT C-3’), and reverse, CpEF1α-NotI-R (5’-ACT GCG GCC GCT TAC TTC TTC TTG GAA GTG G-3)] that provided *Eco*RI and *Not*I restriction sites. The *C. parvum* actin (CpActin) open reading frame was amplified by PCR with a pair of primers [forward, CpActin-SalI-F (5’-ACT GGT CGA CTC ATG AGT GAA GAAGAA ACA C-3’), and reverse, CpActin-NotI-R (5’-ACT GCG GCC GCT TAG AAG CAC TTT CTG TG-3)] that provided *Sal*I and *Not*I restriction sites. The amplified fragments of CpEF1α, and CpActin were digested with *Eco*RI/*Not*I and *Sal*I/*Not*I, respectively, and were cloned into the pGEX-6P-1 vector (GE Healthcare UK Ltd, Buckinghamshire England) by using a DNA ligation kit (TAKARA BIO INC., Shiga, Japan) to express a glutathione *S*-transferase (GST) fusion protein.

### Expression, purification, and concentration of GST fusion proteins

The resulting plasmids and pGEX-6P-1were transformed into competent cells of *E. coli* strain BL21. The recombinant fusion proteins, designated rCpEF1α, rCpActin, and rGST, were expressed following induction with 0.25 mM isopropyl β-D-1-thiogalactopyranoside (Wako Pure Chemical Industries, Ltd., Osaka, Japan). *E. coli* cell cultures expressing recombinant proteins were centrifuged at 5,000 rpm for 30 min, and lysed with lysis buffer [40 mMTris-HCl (pH 7.5), 150 mM NaCl, 1% TritonX-100, complete proteinase inhibitor cocktail (Roche)]. Cell lysates were subsequently centrifuged at 10,000 rpm for 10 min, and the supernatants containing recombinant protein were mixed with a 50% slurry of glutathione-sepharose beads (BD bioscience, NJ, USA) at room temperature for 30 min. The beads were washed with PBS three times and eluted with elution buffer (10 mM glutathione and 500 mM Tris-HCl, pH 8.0).

### Pull-down assay

To identify the parasite proteins that interact with heparin, excysted *C. parvum* sporozoites were lysed with 1% octylglucoside (OGS, Sigma-Aldrich) in PBS overnight at 4 °C^14^, and centrifugation at 10,000 rpm for 25 min. The supernatants were mixed with heparin-agarose beads (Sigma-Aldrich) at 4 °C for 1 h. The beads were then washed with 0.1% OGS in PBS three times.

To confirm the interaction of parasite proteins with heparin, the purified recombinant proteins were mixed with heparin-agarose beads (Sigma-Aldrich) at 4 °C for 1 h. The beads were washed with PBS three times. The beads were then boiled for 5 min in equal volumes of 2 × sample buffer that contained 0.125 M Tris (pH 6.8), 4% SDS, 20% glycerol, and 10% 2-mercaptoethanol.

### Silver staining, mass spectrometry and mascot search

The proteins eluted from the beads described above were separated by SDS-PAGE and then silver stained. The gel was stained by using the Ez stain silver kit (ATTO, Tokyo, Japan). Protein bands were excised from the gels, digested with trypsin and subjected to nano-LC/MS/MS analysis by following the standard protocol using QSTAR XL (Applied Biosystems, CA, USA) and Bio NanoLC (KYA Technologies, Tokyo, Japan). Protein digestion, nano-LC/MS/MS analysis, and the mascot search were conducted by Japan Proteomics (Sendai, Japan).

### Functional enrichment analyses

To functionally categorize the proteins identified in our study, the proteins were assigned to a GO grouping. GO analysis was carried out by using the Gene Ontology Enrichment embedded in the CryptoDB database (http://cryptodb.org/); Fisher’s exact *P* values were used to determine the GO terms that were significant (*P *< 0.05).

### Flow cytometry

The purified rCpEF1α and rGST were concentrated by using Amicon Ultra filter units (50 K for rCpEF1α and 3 K for rGST) (Merck Millipore). The protein concentration was measured by using Protein Quantification Kit-Rapid (Dojindo laboratories, Kumamoto, Japan), and was diluted to 7.5 μM with elution buffer. Semi-confluent HCT-8 cells were washed twice, and treated with 500 μM EDTA in PBS for 5 min at 37 °C. Detached cells were washed with FACS buffer (PBS containing 2% fetal calf serum) by centrifugation at 1,500 rpm. Then 2 × 10^6^ washed HCT-8 cells were incubated with 100 μL of 7.5 μM rCpEF1α or rGST for 2 h at 4 °C, and then washed twice with FACS buffer. The cells were then incubated with 100 μL of rabbit α-GST antibody (Sigma-Aldrich) at 1:1000 dilution for 30 min at 4 °C, washed with FACS buffer containing 2% FCS twice, incubated with 100 μL of Alexa 488-conjugated α-rabbit IgG goat antibody at 1:1000 dilution for 30 min at 4 °C, and washed with FACS buffer again. As a negative control, detached HCT-8 cells which were incubated with neither proteins nor antibodies were also prepared. The sample was then analyzed on BD FACSVerse (BD Bioscience) using BD FACSuite software (BD Biosciences). HCT-8 cells were gated on forward/side-light scatter to distinguish them from debris. Cells (10,000 events) were analysed by Alexa 488 channels.

## Additional Information

**How to cite this article**: Inomata, A. *et al.* Heparin interacts with elongation factor 1α of *Cryptosporidium parvum* and inhibits invasion. *Sci. Rep.* **5**, 11599; doi: 10.1038/srep11599 (2015).

## Figures and Tables

**Table 1:** Heparin-binding proteins of *C. parvum* identified by using nano-LC/MS/MS.

Sample	Protein	MW(Da)	Score^a^	Peptide^b^	Coverage^c^(%)
1	hypothetical protein with a signal peptide	111,402	741	29	35
	conserved hypothetical protein	123,651	442	26	31
	domain KOG1015, transcription regulator XNP/ATRX, DEAD-box superfamily, signal peptide	91,962	429	25	32
	signal peptide, large secreted protein	120,615	327	24	24
	hypothetical protein containing a signal peptide	114,961	136	7	8
	signal peptide, large protein	147,338	50	5	4
	phosphoenolpyruvate carboxylase	130,753	34	1	0
2	acetaldehyde reductase plus alcohol dehydrogenase (AdhE) of possible bacterial origin	94,675	509	25	38
	Eft2p GTpase; translation elongation factor 2 (EF-2)	93,215	501	26	38
	unconventional myosin	91,421	390	22	25
	zincin/aminopeptidase N like metalloprotease	105,843	382	21	25
	heat shock protein 90 (Hsp90), signal peptide plus ER retention motif	89,137	336	19	31
	glycogen phosphorylase	104,146	301	23	27
	KH domain protein	91,196	220	9	11
	Hsp90	82,302	186	13	24
	conserved hypothetical protein	102,374	102	3	4
	CDC48 like AAA ATPase ortholog	90,504	43	2	2
	conserved hypothetical protein	123,651	35	2	1
3	Actin	42,147	401	23	61
	elongation factor 1 alpha	48,131	379	20	62
	phosphoglycerate kinase 1	43,286	274	13	39
	60S ribosomal protein-like, putative	43,224	224	13	44
	hypothetical protein	34,486	198	11	28
	hypothetical protein, signal peptide, predicted secreted protein	40,831	178	8	30
	conserved protein of possible plant or bacterial origin	47,123	65	4	13
	elongation factor EF1-gamma (glutathione S-transferase family)	43,061	47	3	7
	protein kinase, cAMP-dependent, catalytic chain	45,424	44	2	4
	ATP-dependent RNA helicase, putative	46,008	36	2	5
	protein disulfide isomerase, signal peptide, ER retention motif	50,484	35	3	6
	membrane associated thioredoxin	42,072	34	2	6
	26S proteasome regulatory subunit S10b like AAA + ATpase	45,515	33	4	7

Heparin-binding proteins identified throughnano-LC/MS/MS analysis are listed. Proteins whose ion score were >31 are shown.

^a^Protein score. This number reflects the combined scores of all observed mass spectra that can bematched to amino acid sequenceswithin that protein. A higher score indicates a more confident match.

^b^The number of peptides included in each identified protein.

^c^The ratio of the sequence of the identified protein occupied by the found peptides.
